# Direct production of biodiesel from high-acid value *Jatropha *oil with solid acid catalyst derived from lignin

**DOI:** 10.1186/1754-6834-4-56

**Published:** 2011-12-07

**Authors:** Fei-ling Pua, Zhen Fang, Sarani Zakaria, Feng Guo, Chin-hua Chia

**Affiliations:** 1 Universiti Kebangsaan Malaysia, School of Applied Physics, Faculty of Science and Technology, 43600 Bangi, Selangor, Malaysia; 2 Chinese Academy of Sciences, Biomass Group, Laboratory of Tropical Plant Resource Science, Xishuangbanna Tropical Botanical Garden, 88 Xuefulu, Kunming, Yunnan Province 650223, China

**Keywords:** biodiesel, Kraft lignin, *Jatropha *oil, solid acid catalyst

## Abstract

**Background:**

Solid acid catalyst was prepared from Kraft lignin by chemical activation with phosphoric acid, pyrolysis and sulfuric acid. This catalyst had high acid density as characterized by scanning electron microscope (SEM), energy-dispersive x-ray spectrometry (EDX) and Brunauer, Emmett, and Teller (BET) method analyses. It was further used to catalyze the esterification of oleic acid and one-step conversion of non-pretreated *Jatropha *oil to biodiesel. The effects of catalyst loading, reaction temperature and oil-to-methanol molar ratio, on the catalytic activity of the esterification were investigated.

**Results:**

The highest catalytic activity was achieved with a 96.1% esterification rate, and the catalyst can be reused three times with little deactivation under optimized conditions. Biodiesel production from *Jatropha *oil was studied under such conditions. It was found that 96.3% biodiesel yield from non-pretreated *Jatropha *oil with high-acid value (12.7 mg KOH/g) could be achieved.

**Conclusions:**

The catalyst can be easily separated for reuse. This single-step process could be a potential route for biodiesel production from high-acid value oil by simplifying the procedure and reducing costs.

## Background

Recently, biodiesel has gained significant attention as it is a renewable, biodegradable, less pollutant emitting, non-toxic and more environmentally friendly fuel source as compared with the fossil diesel fuel available at present. It is a renewable and biodegradable fuel that consists of fatty acid methyl esters (FAMEs). It is carbon neutral because the carbon content in the exhaust is equal to the amount initially fixed from the atmosphere [1-5]. According to previous reports, the raw materials for biodiesel production account for almost 75% of the total biodiesel cost [3,6]. Therefore, a number of research projects have been carried out using non-edible oils such as *Jatropha *oil or fats, and other waste oils, to reduce the raw material cost. Nevertheless, such oils usually contain a high percentage of free fatty acids (FFAs) that severely affect the biodiesel production process. The high FFA content (>1 wt%) will form soap when a homogenous base catalyst (for example, NaOH) is used, resulting in difficulty in separating products and causing a low biodiesel yield [3,7,8]. Therefore, a two-step process of acid esterification and base transesterification is normally used to convert such oils to biodiesel [9-13]. Production of FAMEs is usually catalyzed by homogenous basic or acidic catalysts such as NaOH, KOH and NaOCH_3 _or sulfuric acid and phosphoric acid [13-16]. However, these homogeneous catalysts create several problems at the end of the reactions, including difficulty in separation of the catalysts, production of pollutants, corrosion of the reactor, sulfur contamination in the biodiesel, and formation of soap [3,17]. In contrast, solid acid catalysts possess advantages over conventional homogeneous acid and base catalysts by being easier to separate from the end products, having comparable catalyst activity and giving a lower amount of pollutants [2]. Recently, wide attention has been given to producing a solid acid catalyst for replacing homogeneous acid catalysts. Previous work has produced carbon-based solid acid catalysts by sulfonating carbonized polymer for hydrolysis [18]. Many studies have been performed using solid acid catalysts for biodiesel production. A good solid acid catalyst should simultaneously catalyze esterification of fatty acids in the oil and transesterification of triglycerides [17,19-22]. Therefore, the use of solid acid catalysts has gained more and more attention in recent years.

Lignin is the second-most abundant natural organic material after cellulose, and the richest aromatic organic biopolymer. It has high carbon content and should be usable as a precursor for activated carbon. Lignin is generally collected from the major waste material from paper mills: black liquor. Waste black liquor lignin can be a low-cost material for the preparation of solid acid catalysts [23,24]. However, there has been little research performed with regard to its application in biodiesel production. Only high-cost carbohydrate-based biomass (for example, starch, glucose) has been used as a raw material to make solid acid catalysts, showing high catalytic activity for biodiesel production from low-qualified oils with high FFAs [10,25].

In this study, we prepared a solid acid catalyst from Kraft lignin by treatment with phosphoric acid, pyrolysis and sulfuric acid, and subsequently it was used as catalyst to synthesize biodiesel from high-acid value *Jatropha *oil. In the biodiesel production process with the catalyst, first, the esterification of oleic acid was studied with an orthogonal experimental design to optimize reaction variables. Various reaction parameters, such as catalyst loading, reaction temperature and oil-to-methanol ratio on the esterification rate were optimized. Under these optimized conditions, crude *Jatropha *oil with high FFAs was directly converted to biodiesel with the solid acid catalyst.

## Results and discussion

### Characterization of solid acid catalyst

**Figure 1:**
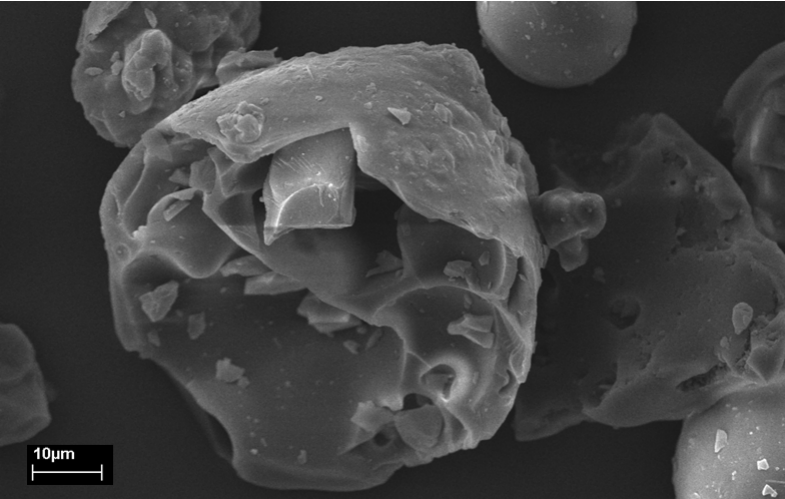
**Scanning electron microscope (SEM) images of raw Kraft lignin**. This shows a typical SEM result for Kraft lignin from Sigma-Aldrich.

The surface morphology of the char was studied by scanning electron microscope (SEM) analysis. Figure 1 shows a typical morphology for Kraft lignin powders obtained from Sigma-Aldrich (Shanghai, China). They have a rounded or semispherical shape with many open volumes on the rough surface. According to a previous report [26], such morphology might be due to the concentration process of extracting lignin from black liquor. Regardless, a spherical shape can be thermodynamically more stable compared to other particle shapes.

**Figure 2:**
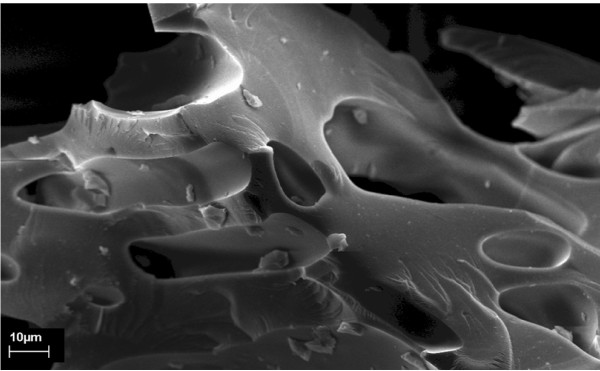
**Scanning electron microscope (SEM) image of Kraft lignin char without chemical activation**. Figure 2 shows SEM images of the pyrolyzed Kraft lignin char without phosphoric acid pretreatment. The surface of char in Figure 2 is smooth and has fewer pores.

**Figure 3:**
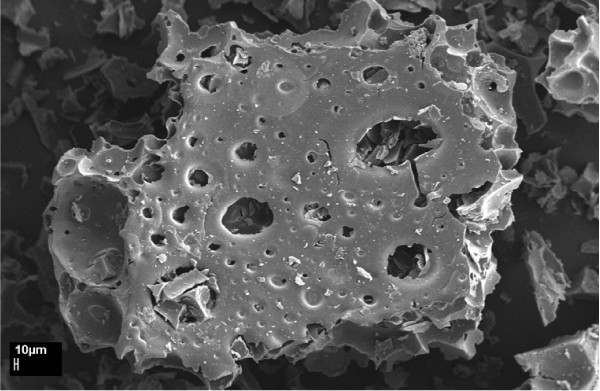
**Scanning electron microscope (SEM) image of Kraft lignin char after chemical activation by H_3_PO_4_**. Figure 3 shows SEM images of the pyrolyzed Kraft lignin char with phosphoric acid pretreatment, respectively. The char pyrolyzed from the phosphoric pretreated lignin is completely different in morphology as compared with the raw Kraft lignin and the char without phosphoric acid pretreatment.

**Figure 4:**
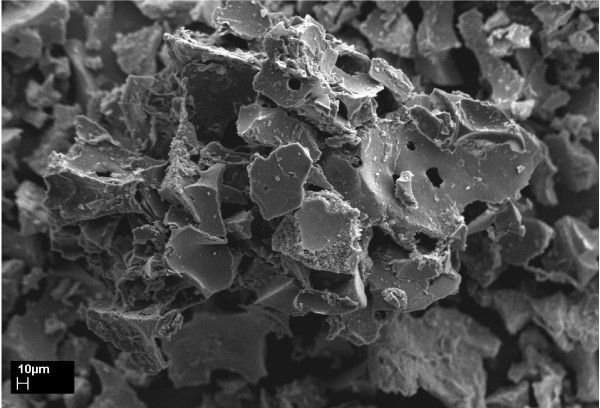
**SEM image of sulfonated Kraft lignin char after phosphoric acid pretreatment and pyrolysis (as catalyst)**. It can be seen that the porosity of the chars (catalyst) was reduced significantly

Figures 2 and 3 show SEM images of the pyrolyzed Kraft lignin char without and with phosphoric acid pretreatment, respectively. The surface of the char in Figure 2 is smooth and has fewer pores. However, it can be seen in Figure 3 that the char pyrolyzed from the phosphoric pretreated lignin is completely different in morphology as compared with the raw Kraft lignin and the char without phosphoric acid pretreatment. Pores developed on the char, produced from phosphoric acid pretreated lignin due to chemical activation before the pyrolysis process. Figure 4 shows an SEM image of the sulfonated Kraft lignin char after phosphoric acid pretreatment and pyrolysis (as catalyst). It can be seen that the porosity of the chars (catalyst) was reduced significantly after the sulfonation process. This can be attributed to the impregnation of SO_3_H groups into the pores of the char.

**Figure 5:**
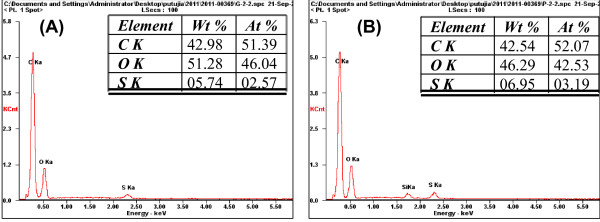
**Energy-dispersive x-ray spectrometry (EDX) spectra of sulfonated Kraft lignin chars (A) without phosphoric acid pretreatment and (B) with phosphoric acid pretreatment (catalyst)**. Figure 5 shows the EDX spectra for the Kraft lignin char with and without acid treatment and the presence of sulfonic group in the char.

Table 1 shows the Brunauer, Emmett, and Teller (BET) method apparent surface area of the Kraft lignin char, pretreated Kraft lignin char and sulfonated char derived from Kraft lignin. The untreated Kraft lignin char gives a surface area of 127.6 m^2^/g. The surface area of phosphoric pretreated char increased dramatically to 654.4 m^2^/g, suggesting that phosphoric pretreatment promoted the formation of highly porous structure [26,27]. The surface area of the Kraft lignin char after sulfonation reduced to 54.8 m^2^/g. The single point adsorption total pore volume of pores that were less than 1246.11 Å width at P/P_o _= 0.98 was also checked. This was reduced from 0.544 cm^3^/g to 0.058 cm^3^/g after the sulfonation process. These results are consistent with the SEM images. The reduction of pores and the surface area might be due to the penetration of acid groups on the surface of porous char. The images reveal the well defined pores on char particles. This might be due to an attack on the structure by the strong acid, resulting in shrinkage of the structure and broken bonds [26]. Figure 5 shows energy-dispersive x-ray spectrometry (EDX) results for the sulfonated char; its S content increased from 5.74 to 6.95 wt% (corresponding to an acid density of SO_3_H increasing from 1.8 to 2.1 mmol/g) after pretreatment with phosphoric acid. Temperature programmed desorption (TPD) was also used to assess surface acidity of the catalyst using an automated chemisorption analyzer (Chembet Pulsar; Quantachrome Instruments, Boynton Beach, FL, USA). The acidity was 0.74 mmol/g, which was estimated as being the total amount of NH_3 _released through TPD per gram of catalyst sample. In another measurement, the sulfonated char had 1.30 mmol/g of acidic sites based on the result from titration. Different methods revealed different acid densities.

**Table 1:** Brunauer, Emmett, and Teller (BET) method analysis on surface area and pore volume

Sample	S_BET _(m^2^/g)	V (cm^3^/g)
Kraft lignin char (only pyrolysis)	127.6	0.062
Pretreated Kraft lignin char (phosphoric acid pretreatment + pyrolysis)	654.4	0.544
Sulfonated Kraft lignin char (catalyst)	54.8	0.058

### Esterification of oleic acid

The activity of the catalyst derived from Kraft lignin was assessed by the esterification reaction. Experiments were conducted according to an orthogonal experimental design, and many batches of tests with different combinations of variables were conducted to optimize the esterification process (Table 2). Analysis of variance (ANOVA) results showed that, among the three factors, the amount of catalyst loading was the most important parameter governing the oleic acid conversion rate, with *P *= 0.0153 and degrees of freedom = 2. The results showed that the more the catalyst loading, the greater the conversion rate. The maximum conversion rate was 96.1% under the optimized conditions, that is, catalyst loading 5 wt% based on weight of oleic acid, molar ratio of oleic acid to methanol (1:12), reaction temperature of 80°C and reaction time of 5 h (Table 3). We repeated the test three times under optimized conditions, and found that the experimental error was less than 0.7%. The catalyst was separated, washed and reused three times; the conversion rate was reduced only to 93.6%. The S content of recovered catalyst was 2.06 mmol/g, analyzed by EDX. Its acid density was reduced by only about 1.9%, showing a slight leaching of SO_3_H groups during reaction.

**Table 2:** Orthogonal experimental design of optimization study for oleic acid esterification

	Experimental variables				
		
**No**.	Catalyst loading (percentage based on weight of oleic acid)	Molar ratio oleic acid:methanol	Temperature (°C)	Reaction time (h)	Oleic acid conversion rate (%)
E1	1	1:6	60	5	71.9
E2	1	1:9	70	5	81.3
E3	1	1:12	80	5	85.6
E4	3	1:6	70	5	91.5
E5	3	1:9	90	5	92.7
E6	3	1:12	60	5	93.6
E7	5	1:6	80	5	93.4
E8	5	1:9	60	5	92.6
E9	5	1:12	70	5	95.8

**Table 3:** Esterification of oleic acid under optimized conditions

Optimized experimental variables				
	
Catalyst loading (based on weight of oleic acid)	Molar ratio oleic acid:methanol	Temperature	Reaction time	Oleic acid conversion rate
5%	1:12	80°C	5 h	96.1%

This result suggests that the solid acid catalyst was highly active and stable. The higher conversion rate of oleic acid is possibly due to the high density of the acid (SO_3_-H) sites from sulfonation in the pores of activated carbon by treatment with phosphoric acid and pyrolysis [24], as confirmed by EDX spectra in Figure 5.

### One-step production of biodiesel from *Jatropha *oil

**Figure 6:**
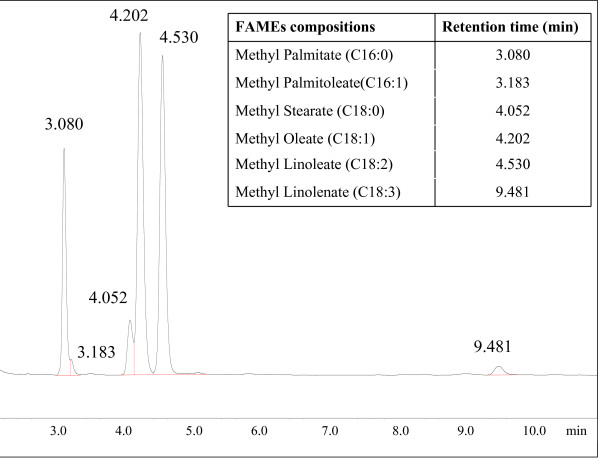
**Gas chromatograph (GC) chromatogram of biodiesel from biodiesel produced from *Jatropha *oil**.

Since the catalyst possessed high catalytic activity in the esterification reaction, we therefore used crude *Jatropha *oil with high-acid value (12.7 mg KOH/g) directly as raw material for biodiesel production without a pretreatment step (or esterification). The conventional method of producing biodiesel from *Jatropha *oil involves esterification and transesterification reactions. However, in this study, a one-step conversion method was selected due to its simplicity and low production costs. The addition of the solid acid catalyst achieved a high biodiesel (FAMEs) yield (96.3%) after a one-step direct conversion of *Jatropha *oil. Figure 6 shows the gas chromatograph (GC) chromatogram for the biodiesel produced from *Jatropha *oil. Phosphoric acid-treated lignin and its chars produced at an elevated temperature possess high porosity and surface area. This allows more surface area for creating SO_3_H acid groups during the sulfonation process and subsequently increases the density of the acid sites (Figure 5).

The solid acid catalyst derived from lignin achieved a higher biodiesel yield as compared with the catalyst derived from carbohydrate (starch) in a previous study, which achieved a 93% yield from waste oils with high FFAs [25]. There was an investigation reporting on preparation of solid acid catalyst from a glucose-starch mixture that obtained 90% biodiesel yield from high FFA content waste cottonseed oil at 80°C [10]. Another advantage of our work is that we used waste lignin to produce the catalyst that is much more environmentally friendly and inexpensive than in other work in the literature.

## Conclusions

In this study, a solid acid catalyst produced from waste Kraft lignin via treatment by phosphoric acid, pyrolysis and sulfuric acid was shown to be useful for esterification and one-step biodiesel production from low-qualified oils due to its high acid density. Assessment for its catalytic activity via esterification proved that it was highly effective in converting oleic acid to ester. This catalyst was further successfully used for biodiesel production, with high yield (96.3%) from non-pretreated *Jatropha *oil. Besides use in biodiesel production, a lignin derived solid acid catalyst may find other applications as a heterogeneous green catalyst.

## Methods

### Materials and catalyst preparation

The analytical grade concentrated sulfuric acid (98%) and phosphoric acid (85%) used for the catalyst preparation was purchased from Chongqing Chuandong Chemical (Group) Co. Ltd., Chongqing, China. Kraft lignin powders were purchased from Sigma-Aldrich, Shanghai, China (produced in St. Louis, Missouri, US). Industrial oleic acid (186 mg KOH/g) (Kermel Corp., Tianjin, China) and *Jatropha *oil (acid value of 12.7 mg KOH/g) from Xishuangbanna Tropical Botanical Gardens [28] were used as model materials for FFAs and crude oil in all experiments, respectively. Dehydrated methanol was from Xilong Chemical Corp., Shantou, China. A 350-ml high-pressure autoclave (FCFD05-30, Yantai Jianbang Chemical Mechanical Co., Ltd., Yantai, China; temperature and pressure can be used up to 320°C and 40 MPa) was used for the esterification and transesterification experiments. A temperature of 60 to 120°C was used in this work.

For the preparation of catalyst, lignin powders were pretreated with concentrated phosphoric acid (85%) [29]. The mixed slurry was left for 1 h at room temperature in air. It was then dried at 105°C for 24 h to allow free vaporization of water, and subsequently pyrolyzed at 400°C for 1 h under nitrogen gas flow [26,29]. The pyrolyzed char was washed several times with hot and cold distilled water to remove residual chemicals, mineral matter and impurities, and oven-dried overnight at 105°C. Sulfonation was carried out at 200°C for 120 min with 1-g char immersed and stirred in 10-ml concentrated sulfuric acid (98%). The sulfonated sample was rigorously washed with hot and cold distilled water to remove any physically adsorbed species until free of sulfate ions. The resulting sample was dried in an oven at 105°C for 48 h and used as the catalyst.

### Esterification of oleic acid

Oleic acid, methanol and the produced solid acid catalyst were loaded and mixed together in an autoclave for the esterification reaction. Experiments were carried out at 60 to 90°C for 5 h. The molar ratio of oil to methanol was 1/6, 1/9, and 1/12. The percentages of catalyst loading were 1 to 5 wt% of oleic acid (Table 2). After reaction, the catalyst was separated from the produced mixture by filtration. Phase separation of filtrate resulted in the isolation of methyl oleate and water. Methanol was removed by distillation of the mixture using a vacuum rotating evaporator.

### One-step conversion of *Jatropha *oil to biodiesel

Similar to the above, non-pretreated crude *Jatropha *oil with methanol and the catalyst were loaded into an autoclave (reaction temperature: 120°C) for esterification and transesterification to biodiesel directly. The reaction parameters were selected based on the results obtained in the esterification reaction of oleic acid. Phase separation of filtrate resulted in the isolation of FAMEs and glycerol. Methanol was removed by distillation of the mixture using a vacuum rotating evaporator. The upper layer of the resulting mixture was recovered as biodiesel (FAMEs).

### Characterizations of solid acid catalyst

The morphology of the solid acid catalyst was examined using SEM (Leo 1450VP). The pore size and pore volume of the as-synthesized product was examined using a BET (Micromeritics ASAP-2020) analyzer operated at -196°C; automatic degas and equilibration interval was 10 s. The elemental composition of solid acid catalyst was examined using EDX. The acid sites on the surface of solid acid catalyst were also determined by the titration method [30]. Solid acid catalyst was added into 0.01 M NaOH aqueous solution and stirred for 2 h at room temperature. Supernatant solution from the centrifugal separation was titrated with 0.01 M HCl.

### Characterization of the products

The acid value of the esterificated products from oleic acid was measured using KOH titration method. The esterification rate (%) was calculated as (Equation 1) [31]:

(1) $$\text{Esterification rate}\left(\%\right)=\left[\left(\text{A}{\text{V}}_{\text{0}}-\text{A}{\text{V}}_{\text{N}}\right)∕\text{A}{\text{V}}_{\text{0}}\right]\times \text{1}00\%$$

Where AV_0 _is the initial acid value of oleic acid and AV_N _is the instant acid value for oleic acid.

Biodiesel obtained from *Jatropha *oil was analyzed by GC (GC-2014, Shimadzu, Japan) with capillary column of Rtx-wax (30 m × diameter 0.25 mm × 0.25 μm). The prepared biodiesel (5 ml) was dissolved in 20-ml dichloromethane and 1-ml internal standard solutions for GC analysis. Heptadecanoic acid methyl ester was used as the internal standard to quantify the yield of esters. The column temperature was 220°C, while the temperatures of the injector and detector were 260°C and 280°C, respectively. Identification of methyl ester peaks was performed by comparing the retention times between the samples and the standard compounds. Methyl esters were quantified by comparing the peak area between the samples and the standard compounds [2,12].

## Competing interests

The authors declare that they have no competing interests.

## Authors' contributions

FLP performed the experiments and drafted the manuscript. ZF supervised the research and helped to draft and revised the manuscript. SZ and CHC helped to revise the manuscript. FG helped with the analyses and some of the experiments. All authors read and approved the final manuscript.
