# Correction: Direct production of biodiesel from high-acid value Jatropha oil with solid acid catalyst derived from lignin

**DOI:** 10.1186/1754-6834-5-66

**Published:** 2012-09-07

**Authors:** Fei-ling Pua, Zhen Fang, Sarani Zakaria, Feng Guo, Chin-hua Chia

**Affiliations:** 1 Universiti Kebangsaan Malaysia, School of Applied Physics, Faculty of Science and Technology, 43600, Bangi, Selangor, Malaysia; 2 Chinese Academy of Sciences, Biomass Group, Laboratory of Tropical Plant Resource Science, Xishuangbanna Tropical Botanical Garden, 88 Xuefulu, Kunming, Yunnan Province, 650223, China

## Correction

We found an important error in our published paper [1]. On page 7, Section: One-step conversion of Jatropha oil to biodiesel, line 3, we wrote a reaction temperature of 120degreesC. The correct temperature should be 220degreesC. The pressure was also omitted from the same reaction description and should be 7.1 MPa.
